# Acute phantosmia as the first manifestaton of brain metastases in a patient with breast cancer. Case report

**DOI:** 10.37796/2211-8039.1641

**Published:** 2025-03-01

**Authors:** Georgios I. Papageorgiou, Nikolaos Skouteris

**Affiliations:** a 2nd Medical Oncology Department, IASO General Clinic, Athens, Greece; b Division of Medical Oncology & Hematopoietic Cell Transplant Unit, Department of Medicine, “Metaxa” Cancer Hospital, 51 Botassi Street, 18537 Piraeus, Greece

**Keywords:** Brain metastases, Cancer, Multidisciplinary, Olfactory, Phantosmia

## Abstract

Phantosmia belongs to the group of olfactory dysfunctions. It is more commonly described in psychiatric conditions and some cases of viral infections, but it has been also rarely described in cancer patients who develop primary or metastatic central nervous system tumors; the early identification of this symptom in this population is crucial, as it could lead to timely diagnosis and treatment through a multidisciplinary approach.

With the current report we present the case of a 60-year-old lady with metastatic breast cancer and without known preexisting brain metastases, who developed acute phantosmia without other neurological deficits; computed tomography of the brain revealed multiple brain metastases, which were attributed to the malignancy, and for which she was effectively treated with whole brain irradiation and antipsychotic as well as anticonvulsant medications. Furthermore, we underline the value of cooperation between the various specialties that could aid in diagnosis and management of this symptomatology.

Phantosmia is an extremely rare symptom in cancer patients, and its appearance should alarm physicians to rapidly investigate a possible progression of disease in the central nervous system. Multidisciplinary approach is needed for the optimal management of these patients.

## 1. Introduction

Phantosmia, or else olfactory hallucinations, is a type of olfactory dysfunction that is more commonly seen in the field of psychiatry and neurology [1]. On the contrary, it is rarely described in cancer patients, where its emergence is consistent with the diagnosis of a primary central nervous system (CNS) tumor [2] or the development of brain metastases in patients with non-CNS tumors [3].

With the current report, we describe the case of a 60-year-old lady with metastatic breast cancer, who was diagnosed with multiple brain metastases after developing acute phantosmia without other accompanying neurological deficits. She received whole brain irradiation (WBRT) and was placed on antipsychotic and anticonvulsant medications, which led to gradual symptomatic improvement; patient’s performance remained unaffected and she was able to continue the treatment plan.

## 2. Case report

A 54-year-old lady was diagnosed with ER (+) PR (+) Her2 (1+) breast cancer with spinal metastases in April 2016, for which she was placed on endocrine therapy and denosumab. She also received Samarium-153-EDTMP in March 2017 due to gradually worsening pain in the known spinal lesions, despite previous conventional radiotherapy to the same lesions in August 2016.

Her disease progressed in October 2018 with multiple lung and liver metastases, so the endocrine therapy was stopped. Between October 2018 and September 2022 our patient received various chemotherapeutic regimens with good tolerance and some short-duration responses, managing to maintain a performance status of 1 most of the time and not to develop brain metastases.

In September 2022, our patient was admitted to our hospital due to development of intermittent incomplete ileus that was attributed to an abdominal hernia. The patient was currently on bevacizumab – capecitabine treatment, suffering from grade II thrombocytopenia at the time of admission, while the last bevacizumab infusion had been given only two weeks before. We discussed her case with the Department of Surgery; both drugs were immediately stopped and the plan was to defer the surgery for 3 weeks and follow a conservative management in the Department of Medical Oncology initially. Her condition gradually improved during the first days of hospitalization and regular surgical reevaluations confirmed the efficacy of the plan.

On the 13^th^ day of hospitalization, the patient seemed quite distressed during the doctors’ ward round. When asked, she stated that she has been experiencing an unpleasant smell of burnt coming from a leakage at the port-a-cath. This discomfort started when she received intravenous paracetamol a few hours before and continued during the infusion of normal saline. Of note, no leakage was confirmed by doctors’ inspection, which raised the suspicion of CNS metastases given the underlying malignancy.

**Fig. 1:**
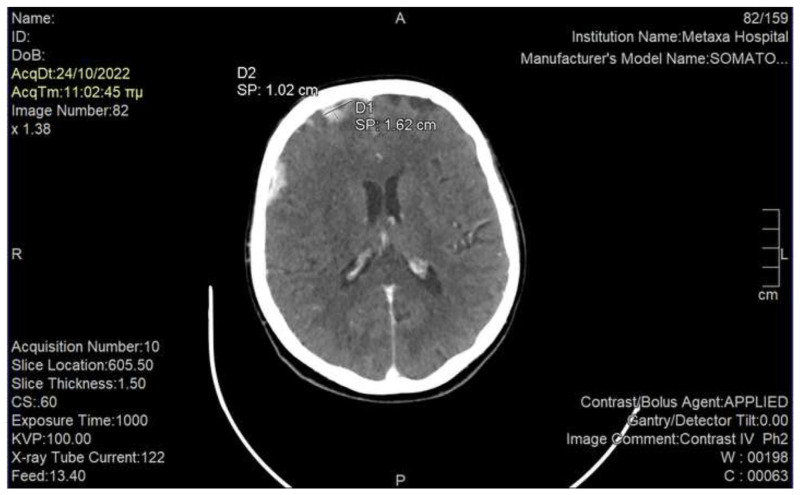
Brain CT showing a 1.6 × 1 cm metastatic brain lesion in the right orbitofrontal cortex, which was considered to cause the patient’s phantosmia.

**Fig. 2:**
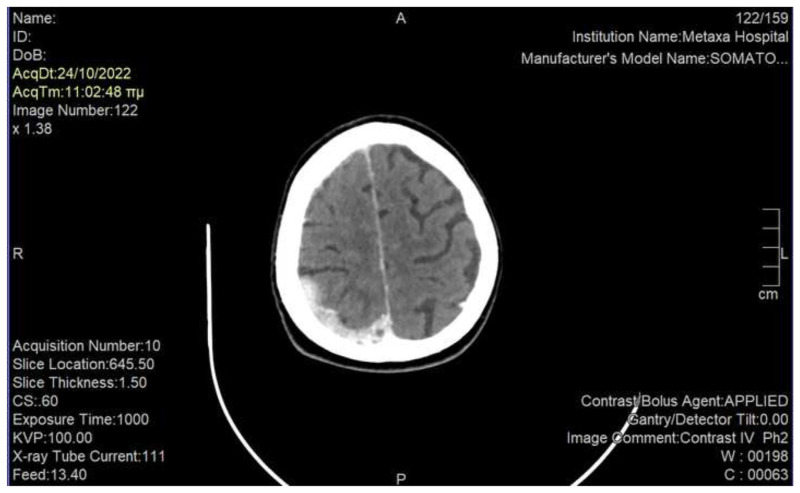
Brain CT showing enhanced leptomeningeal uptake in the right parieto-occipital lobe.

We immediately performed a rapid neurological examination which was normal, and asked for an urgent computed tomography (CT) of the brain. Imaging revealed a 1.6 × 1 cm metastatic brain in the right orbitofrontal cortex among multiple brain metastases, which was thought to be consistent with our patient’s symptomatology [4] (Fig. 1). Notably, there was also evidence of leptomeningeal carcinomatosis in the right parieto-occipital lobe (Fig. 2). We discussed her case in the Tumor Board and decided to proceed rapidly to palliative WBRT. The Neurologists and Psychiatrists suggested to add low-dose antipsychotic medication in order to immediately alleviate the burden of phantosmia until the effects of radiation would occur. Moreover, they agreed to add low-dose anticonvulsant treatment, as literary research indicated that olfactory hallucinations – when presenting as auras – may be the sole manifestation of epilepsy [5].

Given these recommendations, we added olanzapine 5 mg once daily and levetiracetam 500 mg twice daily, while the patient commenced WBRT the day after the onset of the symptom. The episodes and intensity of phantosmia were ameliorated with these modifications, and eventually hallucinations vanished 1 week after WBRT initiation, so we decided to taper olanzapine and continue levetiracetam prophylactically. The patient remains on course to the preplanned surgery without recurrence of phantosmia.

## 3. Discussion

Phantosmia, or else olfactory hallucinations, is an entity that belongs to the group of olfactory dysfunctions [1] and is defined as reporting an olfactory experience in the absence of an emitting factor in the surrounding environment. It is distinguished from parosmia by medical history only, as the definition of the latter requires the presence of an emitting factor [6].

Phantosmia is commonly described in psychiatric disorders – like schizophrenia and psychosis – [1] and in some cases of COVID-19 infection [7]; it is often quite distressing, as it is perceived as an intense unpleasant stimuli – mostly smoky or burnt smells as in our case – although patients generally seem to retain insight that the symptom is not reality-based [1,6]. In our case, though, the patient did not retain insight, which caused distress and contributed additively to the already significant burden of her disease.

There are only some sporadic cases of olfactory hallucinations in cancer patients [3], the majority of which emerge in primary CNS tumors as the first manifestation of disease [2]. It is also known since decades that olfactory auras may represent the only symptom of epilepsy, mostly attributed to pathologic lesions in the temporal lobe [5]. Based on these data, any type of olfactory dysfunction should be investigated with magnetic resonance imaging (MRI) of the brain and electroencephalography [5]. Unfortunately, electroencephalography and MRI were not available at our institution; instead, the patient had a CT that was diagnostic. Our patient’s phantosmia was specifically attributed to a meta-static brain lesion in the right orbitofrontal cortex, which has been shown to mediate conscious olfactory perception; on the contrary, the left orbitofrontal cortex is regarded insufficient to sustain olfaction and may only contribute to processing the emotional content of an odor [4]. The probable leptomeningeal carcinomatosis of our patient was not considered to be causing her symptomatology, as research of the current literature has not provided data to support this correlation.

Surprisingly, a recent retrospective study proved that phantosmia is an underreported adverse event in adolescent and young adult patients receiving proton beam radiation therapy; a possible explanation is direct damage to the olfactory pathways [8]. Moreover, it is known that chemotherapy causes significant transient olfactory dysfunction in a proportion of patients, but deeper qualitative analysis is missing [9].

Of note, there is preclinical evidence that olfactory receptors may serve as oncogenes and drive breast cancer metastasis – especially to the brain – through various metabolic pathways which could be utilized as anticancer targets in the years to come [10].

## 4. Conclusion

Phantosmia in a cancer patient should be distinguished from treatment-related or other causes of parosmia. A rapid neurologic assessment – with electroencephalography – to seek for epileptiform activity – and brain imaging are necessary for prompt diagnosis, as this symptom is more probably consistent with the development of brain metastases in this specific population. In the event of negative brain imaging, a psychiatry consultation could assist diagnosis. Either way, physicians should be alarmed as early recognition and investigation of acute-onset olfactory hallucinations in a cancer patient could preserve a good quality of life with prompt diagnosis and treatment modifications.
